# Annotations

**Published:** 1910-02-26

**Authors:** 


### ANNOTATIONS.

### Kala Azar.

The treatment of kala azar has up to the present been a very hopeless one, but the apparent cure of a case after treatment by atoxyl raised everyone's hopes that perhaps an agent with curative effects for this disease had been found. Whether this be so or not remains to be seen, but a report on "Two Cases of Kala Azar Treated by the Arylarsonates," by Major Harrison and Captain Cumming, in the February number of the *Journal of the Royal Army Medical Corps*, tends to show that at any rate in some cases these drugs appear to be useless. Two soldiers from India were very thoroughly treated by the authors just mentioned, but the downward progress of the disease was not retarded in any way, death eventually taking place in much the same manner as usual in these cases. In the second case, however, an almost complete absence of parasites was found at the autopsy, and this may have been a result of the drug. It is just possible that if the arylarsonate treatment had been begun earlier better results might have been obtained from it, but until this has been done it is premature to speculate. Senega was tried when the patients were almost *in extremis*, the use of this drug having been suggested to the authors by Sir William Leishman, who stated that Captain Ensor had had some promising results from it in the treatment of this disease.

### Educating the Public.

The annual report of the Incorporated Institute of Hygiene, presented at the general meeting of the institute this month, gives encouraging details of the active work which this organisation has been doing to educate the public in the essentials of public and private hygiene. It is a pity that more general support is not given to this excellent society, whose work merits the appreciative attention of the medical profession. The executive is seriously restricted in its labours by want of means, and unless cordial support is extended to the council in its attempt to found a central fund for lectureships and propaganda work there seems a danger that the usefulness of the institute may be seriously curtailed. We would draw special attention to the periodical examinations held under the auspices of the institute in all the leading towns of the United Kingdom. The candidates for these examinations are mainly teachers, and the importance of efficiently educating this class in whose hands very largely rests the instruction of the growing population is not likely to be overestimated. The subjects of the examinations comprise such vitally important matters as the hygiene of motherhood, the rearing of children, cooking and dietetics, the hygiene of the home, home nursing and first aid, physical training, and school hygiene. It is gratifying to find that the number of entrants for these tests—the standard of which is a high one—is progressively increasing, showing that the efforts of the institute have been attended with success. Equally successful have been the popular lectures which members of the institute have held at the large centres. We very strongly favour this method of bringing hygienic essentials to the notice of the public, and we sincerely trust that the profession as a whole will extend its hearty support to these lecture courses. The institute has also co-operated with Viscountess Esher and the St. John's Ambulance Association in providing lectures on First Aid in connection with the Territorial movement, and has actively interested itself in reform measures such as the medical inspection of school children. Last year the Weekly Letter to Members was started—the nucleus of a periodical publication which should do good work. This letter is a helpful and interesting compilation, giving all the information about the working of the institute, institutional and other news, and really instructive paragraphs on hygienic subjects culled from all quarters. What has been done so far, however, is only a step in the right direction. It needs to be followed up by vigorous and sustained effort, particularly in congested districts, before the public can be fully aroused to a proper appreciation of the elementary laws of health.

### Lymphatism.

At a recent meeting of the Section of Anæsthetics of the Royal Society of Medicine an interesting paper on Lymphatism was read by Mr. Bellamy Gardner, which provoked an equally interesting and instructive discussion. The views expressed by the various participants in this debate showed very clearly the wide divergency of opinion that exists on this subject, and emphasise the importance of more thorough investigation and research. None of them were strikingly new. The analogy between the condition described as lymphatism and that known as myasthenia gravis, which was pointed out by Dr. Buzzard, has been alluded to before, but is certainly not one that can be pressed. Many medical men, and still more anæsthetists we believe, are sceptical as to the claims of the former condition for consideration as a definite clinical entity. It is easy enough to find signs of lymphatic hypertrophy in children, and the tendency of late years has been to lay too much stress on post-mortem appearances in cases of sudden death under an anæsthetic, especially in patients whose thymus glands showed some enlargement. How far these post-mortem appearances are to be blamed in causing the fatalities under anæsthetics has still to be investigated, and we cannot accept the evidence hitherto brought forward on the one side as definitely conclusive against the views held by an antagonistic school. For that reason the suggestion that an expert committee should be formed to investigate the whole question and report on it is to be cordially welcomed by anæsthetists as well as by surgeons. Such a committee will have a mass of conflicting evidence to sift and weigh, and will have to undertake experimental work on its own account, but the possibility is that it will be able to throw much light upon a matter which at present is very much veiled in obscurity.

